# A Risk Assessment of the Jaffe vs Enzymatic Method for Creatinine Measurement in an Outpatient Population

**DOI:** 10.1371/journal.pone.0143205

**Published:** 2015-11-24

**Authors:** Robert L. Schmidt, Joely A. Straseski, Kalani L. Raphael, Austin H. Adams, Christopher M. Lehman

**Affiliations:** 1 Department of Pathology and ARUP Laboratories, University of Utah Health Sciences Center, Salt Lake City, Utah, United States of America; 2 Department of Internal Medicine, Division of Nephrology, University of Utah Health Sciences Center, Salt Lake City, Utah, United States of America; University Medicine Greifswald, GERMANY

## Abstract

**Background:**

The Jaffe and enzymatic methods are the two most common methods for measuring serum creatinine. The Jaffe method is less expensive than the enzymatic method but is also more susceptible to interferences. Interferences can lead to misdiagnosis but interferences may vary by patient population. The overall risk associated with the Jaffe method depends on the probability of misclassification and the consequences of misclassification. This study assessed the risk associated with the Jaffe method in an outpatient population. We analyzed the discordance rate in the estimated glomerular filtration rate based on serum creatinine measurements obtained by the Jaffe and enzymatic method.

**Methods:**

Method comparison and risk analysis. Five hundred twenty-nine eGFRs obtained by the Jaffe and enzymatic method were compared at four clinical decision limits. We determined the probability of discordance and the consequence of misclassification at each decision limit to evaluate the overall risk.

**Results:**

We obtained 529 paired observations. Of these, 29 (5.5%) were discordant with respect to one of the decision limits (i.e. 15, 30, 45 or 60 ml/min/1.73m^2^). The magnitude of the differences (Jaffe result minus enzymatic result) were significant relative to analytical variation in 21 of the 29 (72%) of the discordant results. The magnitude of the differences were not significant relative to biological variation. The risk associated with misclassification was greatest at the 60 ml/min/1.73m^2^ decision limit because the probability of misclassification and the potential for adverse outcomes were greatest at that decision limit.

**Conclusion:**

The Jaffe method is subject to bias due to interfering substances (loss of analytical specificity). The risk of misclassification is greatest at the 60 ml/min/1.73m^2^ decision limit; however, the risk of misclassification due to bias is much less than the risk of misclassification due to biological variation. The Jaffe method may pose low risk in selected populations if eGFR results near the 60 ml/min/1.73m^2^ decision limit are interpreted with caution.

## Introduction

Serum creatinine concentration (SCr) is routinely used as a surrogate to evaluate renal function by incorporating the SCr into equations that estimate glomerular filtration rate (eGFR). eGFR calculations are often used to identify and classify patients with kidney failure. Bias in SCr measurements has been a source of concern because of the potential to misclassify patients with respect to renal function.[1–3] Until recently, the lack of traceable standards was a source of variation in SCr measurements. Standardization and harmonization campaigns have reduced calibration bias, [4] but there are still concerns regarding bias due to method non-specificity (i.e., effect of interfering substances).

Two formats are currently available for measuring creatinine: Jaffe assays (picric acid based) and enzymatic assays. The Jaffe assays are more susceptible to interfering substances than the enzymatic method both in frequency and degree of interference, [5] but enzymatic assays are not immune to non-specificity. Several authors have suggested that the Jaffe assay should be abandoned in favor of the enzymatic assay.[6–8]

Though the enzymatic assay is less prone to non-specificity bias than the Jaffe assay, it is considerably more expensive. The reagent list price for one Jaffe assay is approximately $0.30 per test; the cost of the same manufacturer’s enzymatic assay is approximately $2.00 per test. [9] Although the cost savings per test are modest, SCr is a high-volume test. A large hospital laboratory might process 100,000 to 200,000 SCr samples per year. Laboratories could therefore realize substantial cost savings if the majority of SCr measurements could be performed by the Jaffe method. Thus, the choice of SCr method presents a tradeoff between cost and assay performance.

Clinical assay performance can be evaluated from several different perspectives.[10] These perspectives include analytical performance (accuracy, linear range, precision), clinical performance (ability to discriminate disease states), clinical effectiveness (impact on patient outcomes), and cost-effectiveness. These perspectives form a hierarchy in which each level is necessary but not sufficient for performance at the next level (e.g., analytical performance is necessary for clinical performance). Analytical performance is the most basic criteria and is most frequently evaluated in method comparison studies; however, a method should not be evaluated solely on the basis of analytical performance.

Analytical performance is the most fundamental level of assay performance. Method comparison studies generally use the Bland-Altman method to compare the analytical performance of two methods. In this approach, differences (discordances) are plotted against the average result obtained by two methods. These plots are used to determine method bias, limits of agreement, and estimates of the frequency and magnitude of outliers. Bland-Altman analysis places equal weight on all discordances. In practice, some discordant values are more important than others. This occurs because patients are classified with respect to decision limits which, in turn, guide therapeutic decisions. Discordant results that span a decision limit result in misclassification and are more likely to have an impact on patient outcomes. Thus, it is important to evaluate the impact of an assay on classification (clinical performance).

The misclassification rate is an important performance measure; however, misclassifications are only important if they affect patient outcomes. The risk associated with misclassification involves two factors: the probability of an event and the outcome of the event.[11] Risk is defined as the product of these two factors (probability multiplied by outcome). In the context of a diagnostic study, risk is determined by the probability of misclassification and the outcome associated with misclassification. Though the probability of misclassification is important, it is not sufficient to evaluate risk because many misclassifications will not affect outcomes. Thus it is important to incorporate outcomes into assay analysis.

Several studies have compared the Jaffe and enzymatic methods but, to our knowledge, no studies have incorporated outcomes in broad patient populations.[4, 5, 12] Most studies have used spiked samples to demonstrate interferences or have compared results in specialized patient populations such as diabetics.[2–4] Though such studies can show the potential for discordances, they lack generalizability because they are not conducted in broad patient populations. Few method comparisons have been conducted on broad, out-patient populations that include patients from both primary care and specialty medicine clinics. Qiu et al. conducted a method comparison study in patients with chronic kidney disease.[12] They found significant discrepancies in the Modified Diet in Renal Disease (MDRD) eGFR and concluded that the eGFR based on the enzymatic method was superior to eGFR based on the picrate method; however, they based their evaluation on accuracy and did not consider clinical risk. Although these studies were conducted on broad patient populations, the assessment was limited to analytical performance. In summary, no studies have compared the clinical effectiveness of the Jaffe and enzymatic methods in real patient populations. Given the potential economic consequences of assay selection, there is a need for such a comparison.

The Jaffe method could be cost effective in a population where the clinical risk associated with misclassifying patients’ renal status secondary to analytical interferences is low. The risk of misclassification may vary depending upon the relative proportion of patients in the screened population that have interfering substances in their serum (e.g., diabetic versus non-diabetic populations).

Most US laboratories use the Jaffe assay. A recent College of American Pathologists creatinine proficiency testing challenge showed that approximately 70% of the submitted results were based on Jaffe assays.[13] Since most manufacturers offer an enzymatic assay on their instrument platforms, it is reasonable to assume that the predominance of the Jaffe assay is based on a preference for cost over accuracy. Little information is available to assess the clinical impact of using a less specific creatinine for estimating GFR and classifying patients.

This study compared the clinical effectiveness of the Jaffe and enzymatic methods in a broad outpatient population. To that end, we estimated the frequency of discordant eGFR results at defined clinical decision limits in specimens from an outpatient population serviced by a central hospital laboratory and assessed the clinical risk associated with misclassification.

## Methods

### Overview

**Fig 1:**
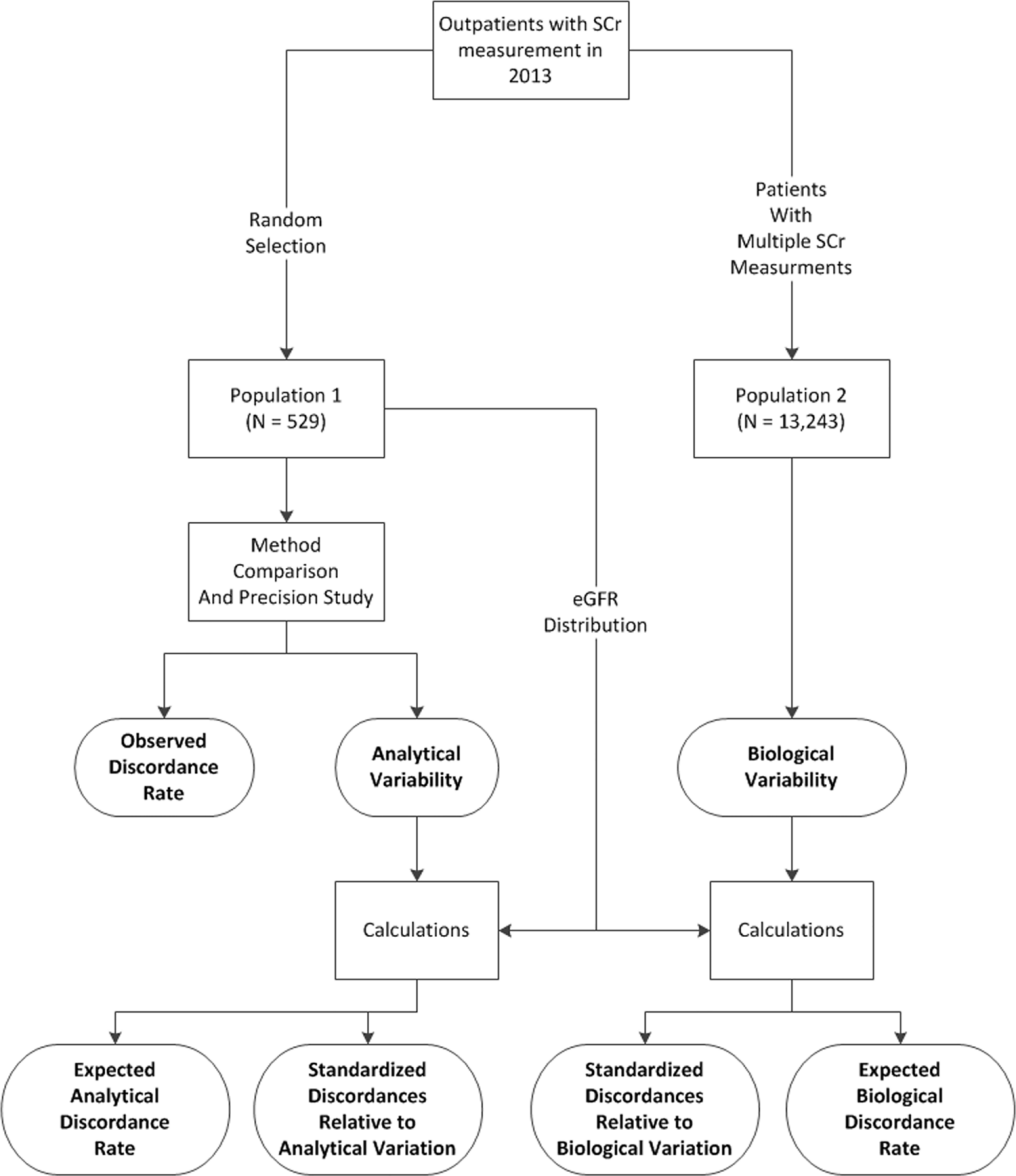
Overview of experimental design. We randomly selected 529 patient samples from outpatient samples that were submitted to the laboratory for SCr measurements (Population 1). This group of samples was used for the method comparison study and to evaluate the analytical precision of the Jaffe and enzymatic methods. We estimated biological variability in the outpatient population by identifying all outpatients who had at least two SCr measurements in 2013 (Population 2). This group contained 13,243 patients with a total of 42,195 SCr measurements. We evaluated the magnitude of discordances (standardized discordances) and the frequency of discordances (expected discordance vs observed discordance).

We determined the frequency and magnitude of discordant eGFR results derived from SCr based on the Jaffe and enzymatic methods and compared the observed discordances against the discordances that would be predicted from analytical and biological variability in eGFR (Fig 1). We focused our study on discordances at clinical decision limits (15, 30, 45 and 60 ml/min/1.73 m^2^). The study was approved by the University of Utah IRB (IRB #00065210). This study was a retrospective database analysis. Patient permission was not obtained. Data was anonymized and deidentified prior to analysis.

### Theoretical background

Results are defined as unconditionally discordant when the values differ. Conditionally discordant results, on the other hand, fall on different sides of a decision limit. This study focuses on conditional discordance but also considers unconditional discordance. We do not always use the terms “conditional” and “unconditional” when the type of discordance is clear from the context. We first discuss misclassification and then discuss the relationship between misclassification and conditional discordance.

Misclassification results from measurement error. There are two sources of measurement error: bias and imprecision. Imprecision is due to a random fluctuation; bias is due to a systematic difference. These two types of error can be distinguished by their response to sample size. Error due to imprecision can be eliminated by increasing sample size; error due to bias cannot be eliminated by increasing sample size.

Misclassification due to imprecision is determined by two factors: the distance of the true result from the decision limit and the imprecision associated with the measurements (S1 Fig). For a single sample, the probability of misclassification increases as the distance between the true result and the decision limit, |*x* − *L*|, decreases. The probability of misclassification of a single sample also increases with the imprecision of the measurement (S1 Fig).

For multiple samples from a patient population, the average probability of misclassification depends on the distribution of the true values relative to the decision limit (S2 Fig). The risk of misclassification increases as the density of the distribution in the region surrounding a decision limit increases. For example, in a population of normal patients (i.e., eGFR > 60 ml/min/1.73 m^2^), the greatest proportion of patients would have values greater than 60 ml/min/1.73 m^2^ and relatively few patients would have low eGFR values. Thus, in such a population, one would expect the misclassification rate to be greater at 60 ml/min/1.73 m^2^ than at 15, 30, and 45 ml/min/1.73 m^2^. The opposite would be expected in a population of patients with chronic kidney disease. The misclassification rate at each decision limit depends on the distribution of eGFR values in the patient population. For this reason, the eGFR distribution of the patient population must be clearly specified in method comparison studies.

Misclassification can also result from bias. Bias occurs when the expected value (average of multiple measurements) differs from the true value. We consider two types of bias: method bias and loss of analytical specificity (LAS). Method bias represents the average difference between methods across multiple samples (e.g., as determined by Bland-Altman analysis). LAS represents the average difference between multiple measurements by two different methods on a single sample.

The measured value can be expressed as a combination of the two types of error:

$$X_{i}=x_{i}+\;e_{i}+b_{i}$$ 1

where *X* is the measured value, *x* is the true value, *e* is the error due to imprecision, and *b* is the bias for sample *i*. We will assume that *e* is normally distributed, $e∼N(0,\sigma_{e}^{2}(x)).$ We expect that the imprecision will vary with the magnitude of the underlying measurement, *x*. We will assume that there is no method bias so that *b*_*i*_ represents LAS. Bias can increase or decrease the probability of misclassification, depending on the direction of the bias relative to the decision limit (S3 Fig). LAS will increase the probability of misclassification with respect to decision limit L when (*L* − *x*_*i*_)(*X*_*i*_ − *x*_*i*_) > 0.

Conditional discordance occurs when observations from two different methods fall on either side of a decision limit. Conditional discordance is related to misclassification because one result must be misclassified to obtain a discordant result. The conditional discordance rate has properties that are similar to the misclassification rate. For an individual sample, the probability of conditional discordance decreases as the distance of the true value from the decision limit, |*x* − *L*|, increases. As with misclassification, the probability of discordance increases with the variability associated with the measurement.

Like misclassification, discordance can be caused by bias or imprecision. The overall discordance rate can be expressed as the sum of discordance due to imprecision and bias:[14]

$$D_{EJ}^{obs}\;=\;{\hat{D}}_{EJ}^{bias}+{\hat{D}}_{EJ}^{prec}$$ 2

where $D_{EJ}^{obs}$ is the observed discordance rate between the enzymatic (E) and Jaffe (J) methods, ${\hat{D}}_{EJ}^{prec}$ is the expected discordance due to imprecision, and ${\hat{D}}_{EJ}^{bias}$ is the estimated discordance due to bias. ${\hat{D}}_{EJ}^{prec}$ can be estimated from the distribution of true values in the sample population and the imprecision of the two methods (J and E).[14] The expected conditional discordance rate due to imprecision, ${\hat{D}}_{EJ}^{prec}$, provides an estimate of the rate at which discordant results would be observed if bias were absent. The discordance due to bias can be estimated by the difference between the observed discordance rate and the discordance attributable to imprecision:

$${\hat{D}}_{EJ}^{bias}=D_{EJ}^{obs}-{\hat{D}}_{EJ}^{prec}$$ 3

If methodological bias is absent, ${\hat{D}}_{EJ}^{bias}$ provides an estimate of the discordance rate caused by LAS. The estimated discordance rates provide a way to compare the observed frequency of discordant results against the frequency of results that would be observed due to imprecision alone and to estimate the impact of LAS.

Discordance rates depend on the variability of the underlying measurement. We used two different measures of variability: analytical variability and biological variability. ${\hat{D}}_{EJ}^{prec}$ based on analytical variability provides an estimate of the discordance rate that would be expected from repeated measurements on a single set of samples taken from the patient population. ${\hat{D}}_{EJ}^{prec}$ based on biological variability provides an estimate of the discordance rate that would be expected from resampling the population and averaging the discordance rate obtained in each sample. The predicted discordance based on analytical variability will usually be less than the discordance rate predicted from biological variability. Thus, given an observed discordance rate, the estimated discordance due to bias (Eq 3) will be higher when based on analytical variability than on biological variability. Both of these estimates provide useful perspectives on discordance rates.

The discordance rate provides a useful performance measure; however, the magnitude of discordance is also important. Given a discordance, one has to evaluate whether the magnitude of discordance is significant. Significance can only be evaluated relative to a measure of variation. As with discordance rates, we evaluated the magnitude of discordances relative to analytical and biological variation.

The difference between readings by two different methods on a single sample is given by:

$$X_{J}-X_{E}=\Delta_{JE}=\;\left(x_{J}-x_{E}\right)+\left(e_{J}-e_{E}\right)+\;\left(b_{J}-b_{E}\right)$$ 4a

$$=\left(e_{J}-e_{E}\right)+\;\left(b_{J}-b_{E}\right)$$ 4b

The expected discordance and variance of the expected discordance are given by:

$$E\left[\Delta_{JE}\right]=\;E\left[\left(e_{J}-e_{E}\right)+\;\left(b_{J}-b_{E}\right)\right]=\;\left(b_{J}-b_{E}\right)$$ 5

$$VAR\left[\Delta_{JE}\right]=\;VAR\left[\left(e_{J}-e_{E}\right)+\;\left(b_{J}-b_{E}\right)\right]=\;VAR[\left(e_{J}-e_{E}\right)]$$ 6

where the subscripts *J* and *E* represent values corresponding to the Jaffe and enzymatic method, respectively. The expressions for the expectation and variance of the difference follow because the true values are independent of the method of measurement (i.e., *x*_*J*_ = *x*_*E*_); the expected value of the error due to imprecision, *e*, is zero; and the bias from a method in an individual sample (*b*_*J*_ *and b*_*E*_) are constants. Eq 5 shows that the average difference obtained from repeated measurements taken by two methods on a single sample provides an estimate of the difference in bias. If the bias in one method is low (e.g. *b*_*E*_ ≪ *b*_*J*_), then *E*[*Δ*_*JE*_] provides an estimate of the bias in the biased method. We also expect the error from imprecision from the two methods to be independent so that

$$VAR\left[\left(e_{J}-e_{E}\right)\right]=\;VAR\left[e_{J}\right]+\;VAR\left[e_{E}\right]=\;\sigma_{J}^{2}\left(x\right)+\sigma_{E}^{2}\left(x\right)=\sigma_{\Delta }^{2}\left(x\right)$$ 7

Discordance was defined as significant if the absolute value of the standardized discordance was greater than 1.96. Standardized discordance, *Z*_*i*_(*L*), for sample *i* at decision limit L, was defined as:

$$Z_{i}\left(L\right)=\;\frac{\left(X_{j}-X_{E}\right)}{\sqrt{\sigma_{J}^{2}\left(x\right)+\sigma_{E}^{2}\left(x\right)}}=\frac{\Delta }{\sigma_{\Delta }\left(x=L\right)}$$ 8

where *X*_*J*_ and *X*_*E*_ are the eGFR observations for the Jaffe and enzymatic methods, L is the decision limit (60, 45, 30, or 15 ml/min/1.73m^2^), and *σ*_*Δ*_(*L*) is the standard deviation of the difference at the decision limit. Standardized analytical discordance was calculated using the analytical variance. Standardized biological discordance was calculated using the biological variation.

Biological variation is often a major component of total variation when samples are taken over time.[15] Estimates of the within person variability range from 4.7% to 16.5%. [16–19] Badrick and Turner estimated that the real change value of the eGFR is 11 ml/min/1.73m^2^ at the 60 ml/min/1.73m^2^ decision limit.[17] The clinical significance of bias (LAS) would depend on the magnitude of the bias relative to the magnitude of the biological variation.

In this study we compare unconditional discordance rates, conditional discordance rates, and the rate of significant conditional discordances.

#### Patient population for method comparison and measurement of analytical variation

The method comparison and analytical variability was based on samples collected at outpatient clinics (Population 1, Fig 1). Five hundred twenty-nine unique outpatient samples were randomly selected from the sample population submitted to the University of Utah hospital laboratory over a 45-day period (7/31/2013 to 9/13/2013). Daily sample sizes were approximately 10% of the eligible outpatient samples.

#### Serum creatinine measurement for method comparison

Sample analyses were performed on the Abbott Architect c8000 analyzer. Each sample was tested by both Jaffe (kinetic alkaline picrate, Abbott Laboratories, Abbott Park, IL) and enzymatic (creatininase, Abbott Laboratories, Abbott Park, IL) methods. After centrifugation, samples were loaded onto Architect sample trays and batch ordered to perform coincident Jaffe and enzymatic testing.

#### Analytical precision of serum creatinine measurements

Analytical precision of the enzymatic and Jaffe assays were determined as follows: Patient specimens were pooled to produce 40 samples at concentrations of 0.28, 0.79, 1.21, 2.73, and 5.08 mg/dL. Each sample was measured daily in duplicate for 20 days. Within-device (analytical) precision was calculated according to established guidelines.[20] The analytical precision at intermediate SCr concentrations was estimated by interpolation. Regression analysis was used to determine the relationship between the coefficient of variation (CV) and SCr.

#### Analytical and biological variation of eGFR measurements

GFR was calculated using the Chronic Kidney Disease Epidemiology (CKD-EPI) equation.[21] The CKD-EPI equation was chosen because we were interested in misclassification at the 60 mL/min/1.73m^2^ decision limit, and the MDRD equation systematically underestimates eGFR above 60 mL/min/1.73m^2^.[22] The CKD-EPI equation also has greater precision than the MDRD equation.[21] Analytical variation of eGFR was estimated from SCr precision using propagation of error calculations (see Appendix 1). Biological variation in eGFR was estimated by selecting all outpatients who had multiple SCr measurements in calendar year 2013 (Population 2, Fig 1). This population consisted of 13,423 patients with 42,195 SCr measurements. (The median time between SCr measurements was 55 days, 5^th^ percentile was 3 days, 95^th^ percentile was 222 days). In this group, SCr was determined by the enzymatic method, and eGFR was estimated by the CKD-EPI equation. The limits of biological variation (within-patient variability) were defined as the 2.5 and 97.5 percentiles of the distribution of consecutive eGFR differences. The biological variability increased with the mean eGFR. For that reason, we determined the biological variability in each of the four zones centered at the 15, 30, 45, and 60 mL/min/1.73m^2^ decision limits (zones were defined as +/- 7.5 mL/min/1.73m^2^ from the decision limit). We used linear regression to estimate the limits of biological variability as a function of eGFR based on the estimates of biological variability in each zone.

#### Method comparison

Bland-Altman plots and orthogonal regression were used to compare calculations (SCr and eGFR) derived from both creatinine methods (enzymatic and Jaffe). We evaluated eGFR discordance at four key clinical decision limits (eGFR = 15, 30, 45, and 60 mL/min/1.73m^2^). [23]

#### Discordant eGFR results

Results were defined as discordant when the eGFR based on the Jaffe and enzymatic methods fell on different sides of a decision limit. The magnitude of discordance was defined as the difference between the Jaffe and enzymatic eGFR values. Discordance was quantified by two different approaches. The overall observed incidence of discordant results was calculated by dividing the number of discordant values by the total number of specimens analyzed. We also estimated regional discordance rates to determine whether the estimated rates varied at the four clinical decision limits. To estimate the number of results at risk for discordance at each decision limit, we defined zones (+/- 7.5 mL/min/1.73m^2^) surrounding each decision limit. The zones were obtained by evenly dividing the regions between the decision limits. The regional discordance was calculated by dividing the number of discordances at a decision limit by the number of specimens contained in the surrounding zone. Zones were defined as follows 15 mL/min/1.73m^2^ (7.5 to 22.5), 30 mL/min/1.73m^2^ (22.5 to 37.5), 45 mL/min/1.73m^2^ (37.5 to 52.5), and 60 mL/min/1.73m^2^ (52.5 to 67.5).

Expected discordance rates were estimated using a published method.[14] In brief, this method estimates the discordance rate that would be expected from consecutive measurements in a patient population based on the underlying imprecision in measurements and the distribution of true values in the population. We calculated expected discordance based on analytical variation and biological variation and compared this to the observed discordance rate seen in our sample (Population 1, Fig 1).

Overall, we compared the observed frequency and magnitude of discordance in eGFR due to differences in the Jaffe and enzymatic method against the frequency and magnitude of discordance that would be expected from analytical variation and biological variation (Fig 1). The magnitude of discordances was compared using standardized differences (relative to analytical or biological variation). The frequency of discordances was evaluated by comparing the observed discordance rate to the expected discordance rate.

#### Risk analysis

We used standard methods for risk analysis.[11] We assessed the probability of misclassification (i.e., the observed and expected misclassification rate) and also assessed the clinical consequences of misclassification. Risk of misclassification was assessed at the 15, 30, 45, and 60 mL/min/1.73m^2^ decision limits.[23] These limits are the recommended limits for classification of patients with respect to chronic kidney disease.

#### Statistical calculations

Correlation analysis was performed using orthogonal (Deming) regression using Minitab 16 (Minitab Corporation, State College, PA). Calculations were performed using Stata 13 (Stata Corporation, College Station, TX). Statistical hypotheses were tested at the 5% significance level.

## Results

### Patient population for discordance analysis

**Fig 2:**
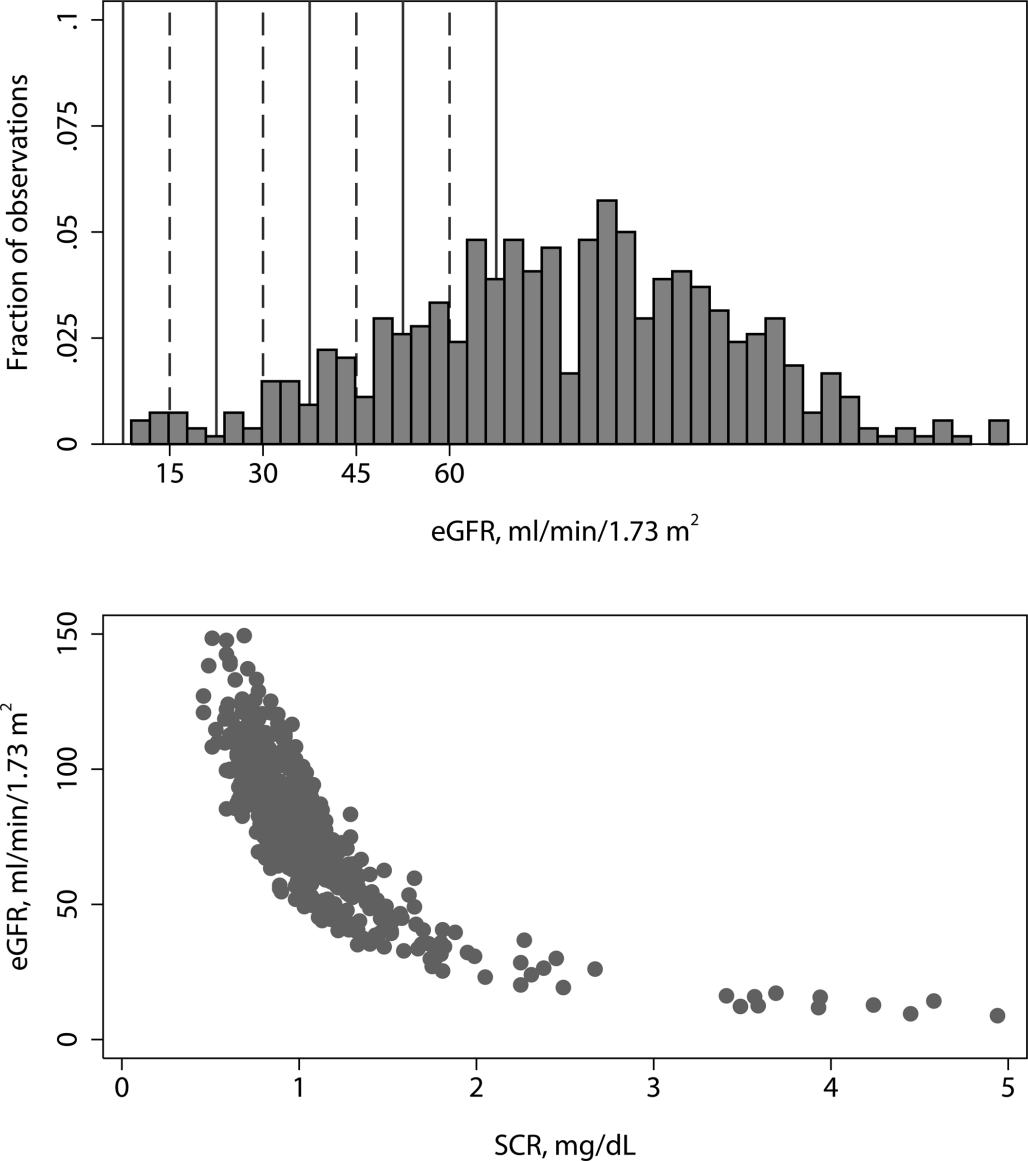
Upper: Distribution of eGFR. The solid lines indicate zones centered at 15, 30, 45, and 60 ml/min/1.73 m^2^. **Lower: Relationship between serum creatinine and eGFR**. eGFR was estimated using the Chronic Kidney Disease Epidemiology (CKD-EPI) equation.

(Population 1, Fig 1): We included 529 patients. We obtained 240 samples from outpatient clinics located at the University of Utah Hospital (mostly specialty medicine clinics) and 289 samples from outpatient clinics located outside the hospital (mostly primary care clinics). The median age was 57 years (range 18 to 94 years). Forty-six percent were male and 2.8% were African American. The median SCr (enzymatic method) was 0.95 mg/dL (95% range: 0.64, 1.81). The median eGFR was 78.8 mL/min/1.73m^2^ (95% range: 32.7, 120.4). The distribution of SCr and eGFR values for the patient population is presented in Fig 2. The zones centered at 15, 30, 45, and 60 mL/min/1.73m^2^ contained 2.4%, 5%, 9.8%, and 17% of the eGFR values, respectively. Sixty-six percent of the eGFR values were greater than 67.5 mL/min/1.73m^2^.

### Analytical variation of creatinine measurements

The Jaffe method had greater precision than the enzymatic method (Table 1). The coefficient of variation of SCr measurements was linearly related to the reciprocal of the SCr concentration. For the Jaffe method, CV = 0.0075 + 0.0066(1/SCr). For the enzymatic method, CV = 0.0113 + 0.0050(1/SCr). Both regressions provided a good fit (R^2^ for Jaffe = 0.88, R^2^ for enzymatic = 0.97). S1 Data

**Table 1:** Comparison of precision of the Jaffe and enzymatic methods for serum creatinine (SCr).

SCr (mg/dL)	Coefficient of Variation (percent)	
	Jaffe method	Enzymatic method
0.28	3.0	2.9
0.79	1.4	1.7
1.21	1.8	1.7
2.73	0.8	1.3
5.08	0.8	1.2

### Analytical variation of eGFR

**Fig 3:**
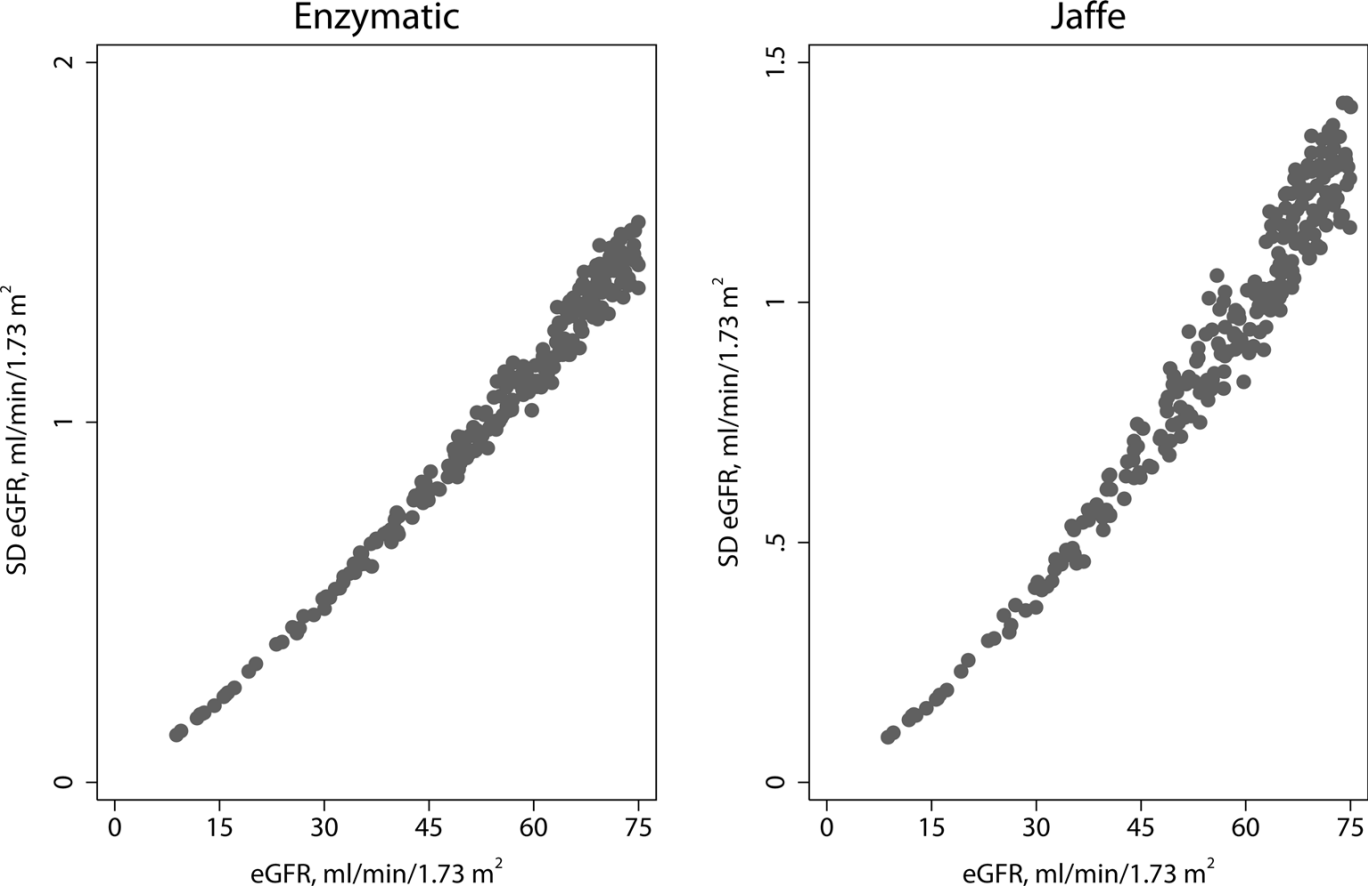
Precision Profile for eGFR Measurements. These Figs show the standard deviation of the eGFR as a function of eGFR for the CKD-EPI method for the enzymatic (left panel) and Jaffe method (right panel). All data (age, sex, and race) are combined.

The precision profile for eGFR is presented in Fig 3. The predicted standard deviation for the difference in eGFR (Jaffe vs enzymatic) ranged from 0.1 mL/min/1.73m^2^ to 3.5 mL/min/1.73m^2^ at eGFR levels of 8 and 126 mL/min/1.73m^2^, respectively.

### Correlation of creatinine measurements

**Fig 4:**
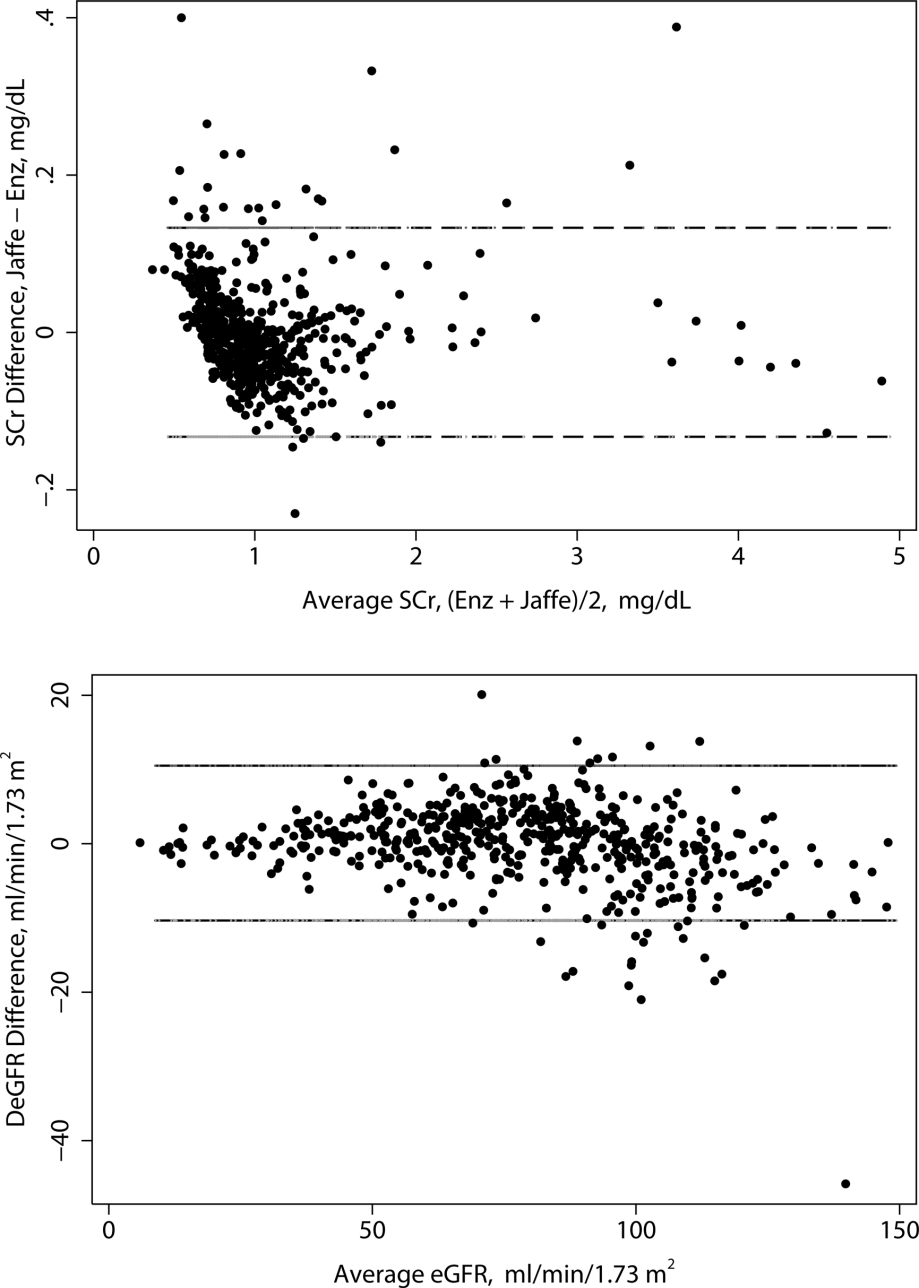
Bland-Altman plots for creatinine and eGFR. Upper panel: Creatinine. Lower panel: eGFR.

The Bland-Altman plot (Fig 4) had a mean of 0.0, a lower bound of -1.32 and an upper bound of 1.33 mg/ dL. Orthogonal (Deming) regression showed no significant difference between the Jaffe and enzymatic methods. The slope was 1.006 (95% CI: 0.998, 1.103) and the intercept was -0.005 (95% CI: -0.015, 0.006). S2 Data

### Correlation of eGFR measurements

The Bland-Altman plot for eGFRs is presented in Fig 4. The mean, lower, and upper limits for the CKD-EPI Bland Altman plot are 0.1, -10.3, and 10.5 mL/min/1.73m^2^, respectively. There was a statistically significant difference between eGFRs based on the Jaffe and enzymatic eGFR. The intercept for the Jaffe-enzymatic regression was 2.66 (95% CI: 1.35, 3.98) and the slope was 0.97 (95% CI: 0.95, 0.99).

### Biological variation of eGFR

**Fig 5:**
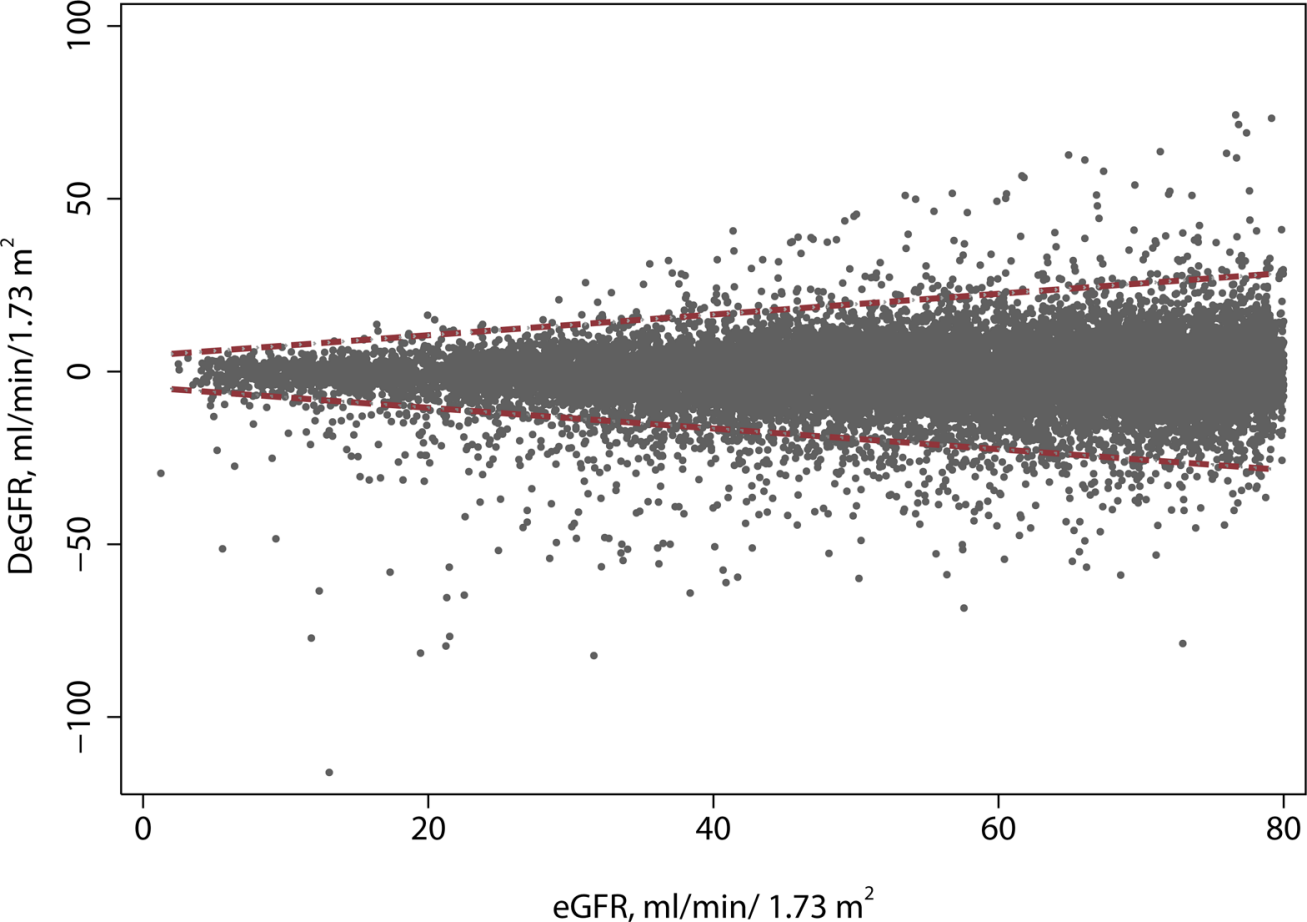
Biological Variability of eGFR. Each point represents the difference between consecutive eGFR measurements in one patient. The dashed line represents the estimated limit of biological deviation (95% confidence limits).

The biological variation of eGFR (Population 2, Fig 1) increased with the mean eGFR. The limits of biological variation were +/- 8, 13.5, 18, and 21.5 ml/min/1.73 m^2^ at the 15, 30, 45, and 60 ml/min/1.73 m^2^ decision limits, respectively (S4 Fig). The limit of biological variation, *L*_*B*_, was approximated by the linear relationship, *L*_*B*_ = 4.0 + 0.3*eGFR which encompassed 97% of the deviations (Fig 5). In this population, the median time between SCr measurements was 55 days (5^th^ percentile was 3 days, 95^th^ percentile was 222 days). On average, the eGFR was stable over time. The eGFR decreased, on average, by 0.0036 ml/min/1.73 m^2^ per day or 1.3 ml/min/1.73 m^2^ per year. S3 Data

### Analysis of unconditional discordance

**Fig 6:**
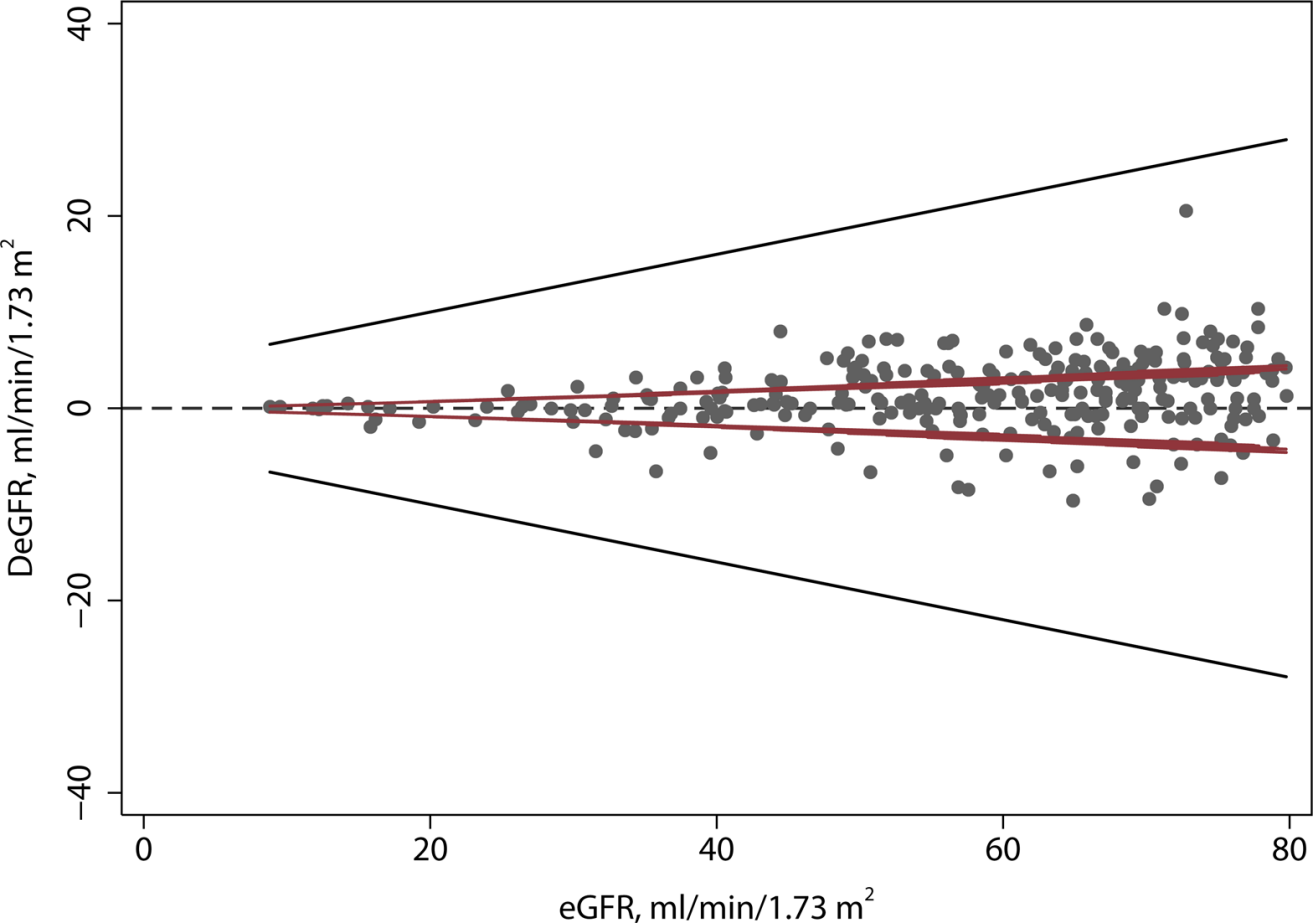
Comparison of analytical variation, biological variation and unconditional discordance. The inner lines (maroon) are the limits of analytical variation and the outer (black) lines are the limits of biological variation. Forty-two percent of the discordances exceed the limits of analytical variation. None of the discordances exceed the limits of biological variation.

The magnitude of the eGFR differences frequently exceeded the magnitude of differences that would be expected from analytical variation. Forty-two percent (227 of 529) of the eGFR differences were significant (i.e., exceeded two standard deviations) relative to analytical variability (Fig 6). The magnitude of eGFR differences were always less than the limits of biological variability, *L*_*B*_. Thus, none of the observed discordances were significant relative to biological variability.

### Comparison of conditional discordance relative to analytical variability

**Fig 7:**
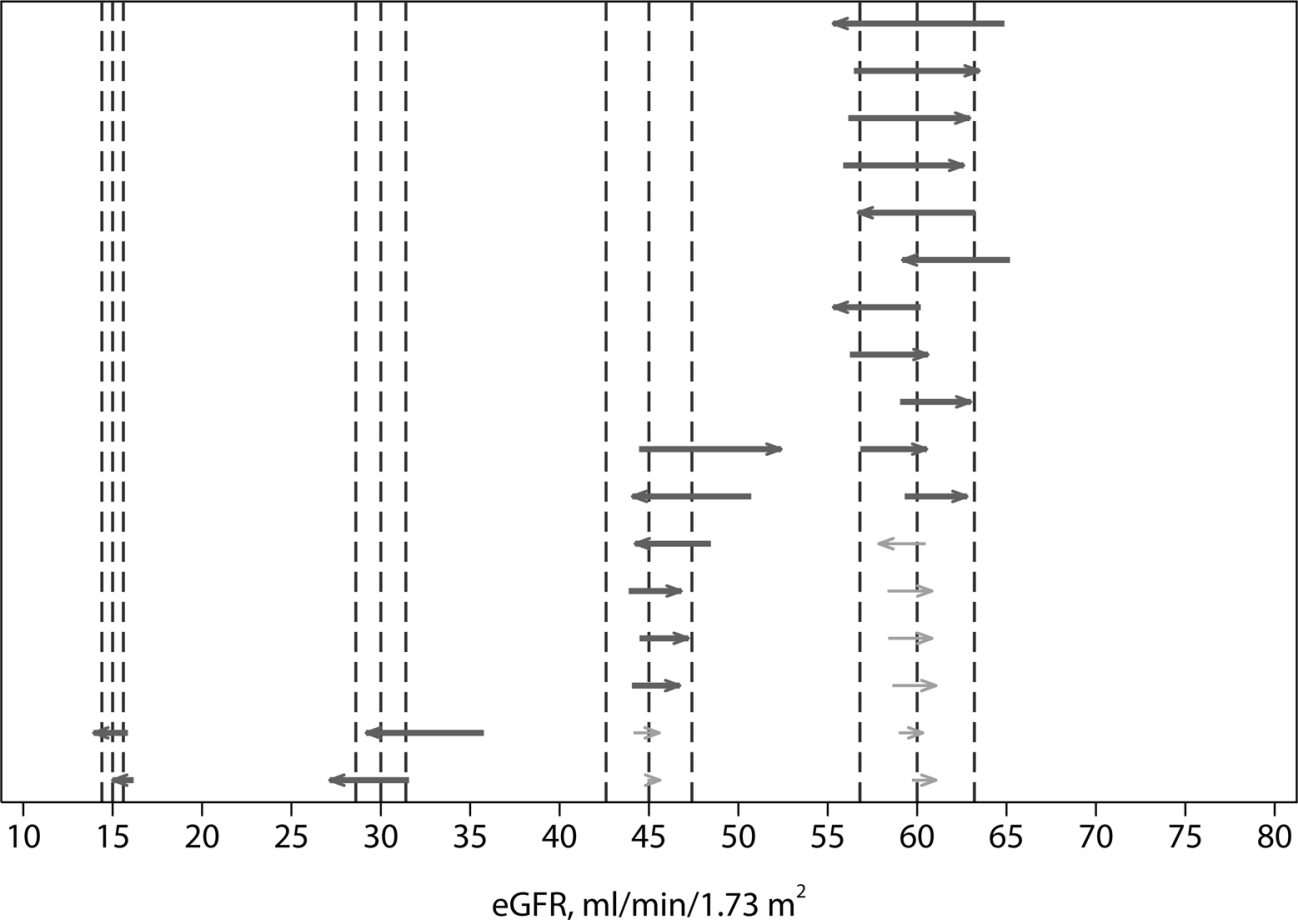
Comparison of observed discordances to analytical variation. Each line represents the difference between the Jaffe result and enzymatic result. The vertical lines indicate the decision limit and two standard deviations of the difference (Jaffe-enzymatic) due to measurement imprecision at the decision limit. The arrows are directed from the enzymatic result toward the Jaffe result (the Jaffe result is greater than enzymatic when arrows point from left to right). Heavy lines indicate statistically significant discordances (i.e., greater than two standard deviations of the analytical variation) and light lines indicate nonsignificant discordances.

Overall, there were 29 (5.5%) conditionally discordant CKD-EPI observations (Fig 7, Table 2). Twenty-one of these 29 discordant observations (72.4%) were significant relative to the analytical variation (i.e., greater than two standard deviations of the estimated analytical precision of the difference). The observed discordance rates were higher than the expected discordance rates at each decision limit (Table 3) and the overall observed discordant rate was (5.3%) was significantly greater than the expected discordance rate (z = 4.6, p < 0.001). There was no significant difference between the discordance rates in each of the zones centered at 15, 30, 45, and 60 mL/min/1.73m^2^; however, there was considerable imprecision in the rate estimates at the lower eGFR decision limits (Table 3).

**Table 2:** Discordance rate by eGFR decision point.

Discordance Type	Decision Limit	Zone Range	Number of Specimens In Zone (%)	SD	ND	Observed Discordance Rate (95% CI)
AD	60	52.5–67.5	92 (17.0)	NA	17	18.5 (11.1,29.9)
	45	37.5–52.5	53 (9.8)	NA	8	15.1 (6.7, 27.6)
	30	22.5–37.5	27 (5.0)	NA	2	7.4 (1.0, 24.2)
	15	7.5–22.5	13 (2.4)	NA	2	15.4 (1.9, 45.4)
	All	0–100	529	NA	29	5.3 (3.6, 7.6)
ASD	60	52.5–67.5	92 (17.0)	1.6	11	12.0 (6.1, 20.4)
	45	37.5–52.5	53 (9.8)	1.2	6	11.3 (4.2, 23.0)
	30	22.5–37.5	27 (5.0)	0.7	2	7.4 (1.0, 24.2)
	15	7.5–22.5	13 (2.4)	0.3	2	15.4 (1.9, 45.4)
	All	0–100	529	NA	21	3.9 (2.4, 5.9)
BSD	60	52.5–67.5	92 (17.0)	11.2	0	0 (0.0,3.9)
	45	37.5–52.5	53 (9.8)	9.1	0	0 (0.0, 6.7)
	30	22.5–37.5	27 (5.0)	7.1	0	0 (0.0, 12.8)
	15	7.5–22.5	13 (2.4)	4.3	0	0 (0.0, 24.7)
	All	0–100	529	NA	0	0 (0.0,0.6)

We calculated the discordance rate for each zone by dividing the number of discordances at a decision limit by the number of samples at risk of discordance (i.e., samples with eGFRs within 7.5 mL/min/1.73m2 of the decision limit). Significant discordances were defined as those with differences greater than two standard deviations of the difference due to analytical imprecision or biological variability. SD = standard deviation, ND = number of discordant observations (i.e., Jaffe and enzymatic measurements on either side of the decision limit), NA = not applicable. Standard deviations represent analytical variation (middle group) or biological variation (bottom group). AD = All discordances. ASD = Analytically significant discordances. BSD = Biologically significant discordances.

**Table 3:** Analysis of expected discordance rates.

Decision Limit	N	Observed Discordance Rate	Expected Discordance Rate		Estimated Discordance Rate Due to Bias	
			Analytical Variability	Biological Variability	Analytical Variability	Biological Variability
60	17	0.0321	0.011	0.163	0.0211	NA
45	8	0.0151	0.006	0.092	0.0091	NA
30	2	0.0038	0.003	0.032	0.0008	NA
15	2	0.0038	0.003	0.007	0.0008	NA
Total	29	0.0548	0.0230	0.294	0.0318	NA

The observed discordance rate (based on 529 observations) is compared to the discordance that would be expected from imprecision alone based on analytical variability and biological variability. NA = not applicable.

The discordances showed no consistent direction. Eighteen of 29 (62%) of the total CKD-EPI discordances were positive (i.e., Jaffe value > enzymatic), and 11 of 21 (52%) of the significant CKD-EPI discordances were positive (Fig 7). The difference between negative and positive discordances was not statistically significant (minimum p value over all decision limits = 0.09).

The observed discordance rate at decision limits was greater than the expected discordance rate. The overall expected discordance rate due to analytical imprecision was 2.3% (Table 3). The observed discordance rate was 5.3%.

### Comparison of conditional discordance to biological variation

None of the discordances were significant relative to biological variation at any decision limit (Table 2, S5 Fig). The observed discordance rate was lower than the expected discordance rate due to biological variation (Table 3).

### Risk analysis

The greatest potential for clinical impact of misclassification occurs at the 60 mL/min/1.73m^2^ decision limit where patients without other clinical evidence of kidney disease risk misdiagnosis of CKD (Table 4).

**Table 4:** Risk assessment for Jaffe method.

	eGFR Decision limit mL/min/1.73m2				Total
	15	30	45	60	
Discordance Rate, CKD-EPI	0.37(0.04, 1.33)	0.37(0.04, 1.33)	1.49(0.64,2.90)	3.13(1.84,4.99)	5.37(3.62,7.62)
Significant, CKD-EPI	0.37(0.04, 1.33)	0.37(0.04, 1.33)	1.11(0.41, 2.40)	2.04(1.02, 3.61)	3.89(2.42, 5.88)
Diagnosis Below Limit	Stage 5 CKD	Stage 4 CKD	Stage 3b CKD	Stage 3a CKD	
Diagnosis Above Limit	Stage 4 CKD	Stage 3b CKD	Stage 3a CKD	No CKD or Stage 2 CKD*	
Management Below Limit	Prepare for dialysis and/or kidney transplantation(monthly visits and lab tests)	Very frequent clinic visits & lab tests (every 3 months)	Frequent clinic visits & lab tests (every 3–6 months)	Periodic clinic visits (every 6–12 months), further testing, nephrology referral, lower blood pressure goal in some cases	
Management Above Limit	Prepare for dialysis and/or kidney transplantation	Frequent clinic visits (every 3–6 months)	Frequent clinic visits (every 6–12 months)	None unless other evidence of kidney injury	
Probability of Mismanagement	Low	Low	Low	Low-Moderate	
Overall Risk	Low	Low	Low	Low	

*if other evidence of kidney injury (i.e., proteinuria, glomerular hematuria, kidney cysts)

eGFR = estimated glomerular filtration rate. CKD = chronic kidney disease.

## Discussion

Our study estimated the impact that adopting the Jaffe method for SCr measurement would have on patient care in a representative outpatient clinic population. We focused on discordance at clinical decision limits (conditional discordance) because these limits are used to classify patients and direct therapy which can affect patient outcomes. We found that the conditional discordance rate was greater than would be expected from analytical variation and less than would be expected from biological variation. The magnitude of the discordances was often significant relative to analytical variation but was insignificant relative to biological variation.

We observed no systematic difference between the methods (average difference between the Jaffe and enzymatic SCr was zero). Therefore, the observed discordances are most likely due to a combination of analytical variation and bias. If we assume that all the discordant observations are attributed to the Jaffe method and that all discordant results represent kidney function misclassifications, then adoption of the Jaffe method would result in an apparent increase of approximately 5.4% in misclassifications. However, we estimate that approximately 28% of the discordant observations were due to analytical imprecision in the eGFR. Thus, the incremental increase in analytically significant misclassification would be approximately 4%. Since only a portion of the discordances can be attributed to the Jaffe method, 4% represents an upper bound on the analytically significant misclassification rate.

Analytical variation provides a stringent criterion with which to evaluate discordance. Although this criterion provides a means to estimate the magnitude and frequency of LAS, analytical variability is much less than the variation that would be seen in clinical practice. The rate and magnitude of discordance that could be attributed to LAS is less than the rate and magnitude of discordance that would arise from daily biological variation in patients. Thus, even if all LAS were attributed to the Jaffe method, it is not clear that the Jaffe method would have a clinically significant impact on misclassification.

Although misclassifications increase the risk of patient harm, the overall risk depends on the likelihood that a misclassification would result in a change in management that would have significant consequences for the patient. The overall risk is determined by the product of the probability of misclassification and the consequence of misclassification.[11] These two factors vary by decision limit so the overall risk varies by decision limit. A misclassification will not lead to harm if the consequences of misclassification are small. A misclassification will not necessarily lead to harm even when the consequences are significant. For example, the expected consequence would be reduced if it was likely that the misclassification would be discovered (i.e., repeat testing, ancillary information) before a change in management. Therefore, accuracy alone is insufficient to evaluate the suitability of an analytical method. We used a risk-analysis framework because it incorporates both the rate and consequences of misclassification.

We assessed the risk associated with misclassification at four decision limits. We evaluated the probability of a misclassification, the impact of the misclassification on patient management, the consequences for the patient, and the likelihood that a misclassification would lead to a change in management. Misclassifications using the Jaffe method at the 15, 30, and 45 mL/min/1.73m^2^ cutoffs were infrequent and the magnitude of the discordances was less than 8 mL/min/1.73m^2^ (range -6.6 mL/min/1.73m^2^ to 7.9 mL/min/1.73m^2^). Therefore, the diagnosis of CKD would not be questioned as long as the eGFR had been low for ≥ 3 months. In general, the effect of misclassification carries less risk at these cutoffs, even at the 15 mL/min/1.73m^2^ threshold when dialysis may be considered since eGFR is not the only factor that determines when dialysis is necessary. More often, dialysis begins when malaise, nausea, weight loss, hypervolemia, hyperkalemia, and acidosis are difficult to manage in the setting of very low eGFR (usually < 10 mL/min/1.73m^2^). Other management issues to consider with very low eGFR include the timing of arteriovenous fistula creation for hemodialysis, placement of peritoneal dialysis catheter, or preemptive kidney transplantation. These are not usually considered until eGFR is < 20 mL/min/1.73m^2^, where the difference between the Jaffe and enzymatic methods is quite small. Overall, the risk of using the Jaffe method over the enzymatic method is low, particularly for those with lower eGFR.

The most frequent incidence of significant misclassifications in our data set that could be attributed to use of the Jaffe assay (≤ 2%) occurred at the 60 mL/min/1.73m^2^ cutoff—the threshold for CKD diagnosis assuming there is no other evidence of renal disease.[23] The main effect of misclassification at an eGFR of 60 mL/min/1.73m^2^ is in terms of CKD diagnosis. Misclassification of a patient to an eGFR < 60 mL/min/1.73m^2^ would inappropriately diagnose CKD which could lead to further testing, nephrology referral, and patient anxiety, although it is important to remember that the diagnosis of CKD based on low eGFR alone requires persistence of the low eGFR for at least three months.[24, 25] On the other hand, misclassification of a patient to an eGFR > 60 mL/min/1.73m^2^ will fail to diagnose CKD, which is a major risk factor for cardiovascular disease and death.[26] Furthermore, guidelines recommend lower blood pressure targets for people with CKD (i.e., systolic blood pressure < 140 mm Hg).[27] Failing to diagnose CKD may not prompt treatment of blood pressure to these targets. Finally, nephrology referral and other diagnostic tests may not be considered.

Discordances at the 60 ml/min/1.73m^2^ limit pose the greatest risk because patients who are misclassified above 60 may be lost to followup when they have kidney disease. Thus, disease could progress. Overall, our results suggest that the rate of such misdiagnosis due to biologically significant variation is quite small. The risk of misdiagnosis by eGFR based on the Jaffe method could be reduced by excluding patients with high likelihood of interference (e.g., known or new diabetics) or by reflexing all higher risk eGFR observations (i.e., those near the 60 ml/min/1.73m^2^ limit) for testing by another method, such as with cystatin C, which when combined with SCr in the CKD-EPI equation is more accurate in persons with more preserved renal function.[23]

We estimated that the within-person variability is approximately 37% which corresponds closely to the estimates provided by Klee et al. who obtained estimates of 33% in an outpatient population.[19] Selvin et al. found that the within-person eGFR was 13.2% and that the discordance rate was approximately 17% at the 60 ml/min/1.73m^2^ limit.[16] Another recent study performed showed that 52% of patients with an initial eGFR below the 60 ml/min/1.73m^2^ limit were confirmed to have chronic kidney disease after a second eGFR measurement.[28] Our results also suggest that eGFR results near the 60 ml/min/1.73m^2^ limit should be repeated to reduce the chance of misclassification.

Our analysis has several limitations. Most importantly, we did not compare the Jaffe and enzymatic methods to a gold standard. Thus, our analysis only provides relative error rather than absolute error. However, our risk analysis is not dependent on absolute error. We assumed that all errors were due to the Jaffe method and thereby obtained an upper limit to the error rate of the Jaffe method. Using this upper limit, we found that the Jaffe method presents relatively low risk to patients. The 4% error rate may seem unacceptable; however, it is the clinical risk (probability of misclassification x consequence of misclassification) that is important. Our analysis suggests that the risk associated with the Jaffe method may be acceptable, particularly in view of the high cost associated with the enzymatic method and the frequency of expected discordances secondary to biological variability. This may justify the use of the Jaffe method for outpatient screening.

## Conclusion

The Jaffe method is less expensive than the enzymatic method but is also more susceptible to interferences. We estimate that the misclassification rate associated with the Jaffe method would be approximately 4%. Misclassifications would occur primarily at the 60 ml/min/1.73m^2^ decision limit. Although the Jaffe method would increase misclassifications, the potential for mismanagement and patient harm is relatively low because diagnosis of CKD is not based on eGFR alone.

## Appendix 1

### Calculation of the Measurement Precision of eGFR. [29]

The equations for eGFR depend on age, sex, race, and SCr. Therefore, the variability in eGFR due to imprecision in SCr depends on age, sex, and race.

The CKD-EPI equation is:

$$eGFR_{CKD}=\;141{\left[\text{min}\;\left(\frac{SCr}{\kappa },1\right)\right]}^{\alpha }\;{\left[\text{max}\;\left(\frac{SCr}{\kappa },1\right)\right]}^{-1.209}\;(0.993^{Age})K_{3}K_{4}$$ A.1

where $\alpha =\{\begin{array}{c} -0.329\;if\;female\\ -0.411\;otherwise \end{array},\;\kappa =\{\begin{array}{c} 0.7\;if\;female\\ 0.9\;otherwise \end{array},\;K_{3}=\{\begin{array}{c} 1.018\;if\;female\\ 1.0\;otherwise \end{array},\;K_{4}=\{\begin{array}{c} 1.159\;if\;black\\ 1.0\;otherwise \end{array}$

Eq A.1 can be written as:

$$eGFR_{CKD}=A{\left(\frac{SCr}{\kappa }\right)}^{B}\;0.993^{Age}$$ A.2

Taking the derivative of A.1 with respect to SCr gives:

$$\frac{\Delta eGFR_{CKD}}{\Delta SCr}=AB\left(\frac{1}{\kappa }\right){\left(\frac{SCr}{\kappa }\right)}^{B-1}\;0.993^{Age}$$ A.3

Multiplying both sides by *ΔSCr* and dividing by *eGFR*_*CKD*_ gives:

$$\frac{\Delta eGFR_{CKD}}{eGFR_{CKD}}=B\frac{\Delta SCr}{SCr}$$ A.4

or:

$$\Delta eGFR_{CKD}=B\frac{\Delta SCr}{SCr}eGFR_{CKD}$$ A.5

## Supporting Information

S1 Fig Impact of bias and precision on the probability of misclassification. Each panel shows the distribution of measurements centered on the true value. The width of the distribution depends on the variation of the measurement. The decision limit is 60 for all three cases. The shaded area indicates probability of misclassification given the distance between the true value and decision limit and the precision.
(TIF)

S2 Fig Impact of the eGFR distribution on misclassification. The graph shows the eGFR distribution for two different hypothetical patient populations. The upper panel represents a relatively healthy population in which the majority of eGFR values are greater than 60 ml/min/1.73 m^2^. In this population, the misclassification rate will be greatest at 60 ml/min/1.73 m^2^. The lower panel represents a specialized population with kidney disease. In this population, the misclassification rate will be greatest at 30 ml/min/1.73 m^2^.
(TIF)

S3 Fig Impact of bias on misclassification. The true result is indicated by a lower case x and the biased value is indicated by an upper case X. The decision limit is indicated by L. Due to imprecision, the observed values will form a distribution around the mean value. In the upper panel, bias moves the mean toward the decision limit and increases the probability of misclassification. In the lower panel, bias moves the mean away from the decision limit and decreases the probability of misclassification.
(TIF)

S4 Fig Limits of biological variation as a function of eGFR. The lower limit is the 2.5^th^ percentile and the upper limit is the 97.5^th^ percentile of observations.
(TIF)

S5 Fig Comparison of observed discordances to biological variation. Each line represents the difference between the Jaffe result and enzymatic result. The vertical lines indicate the decision limit and two standard deviations of the difference (Jaffe-enzymatic) due to measurement imprecision at the decision limit. The arrows are directed from the enzymatic result toward the Jaffe result (the Jaffe result is greater than enzymatic when arrows point from left to right). Heavy lines indicate statistically significant discordances (i.e., greater than two standard deviations of the biologic variation) and light lines indicate nonsignificant discordances (there are no heavy lines in this graph).
(TIF)

S1 Data Precision Data. (XLSX)

S2 Data Method Comparison Data. (XLSX)

S3 Data Biological Variation Data. (XLSX)

## References

[1] Myers GL. Standardization of serum creatinine measurement: Theory and practice. Scandinavian Journal of Clinical and Laboratory Investigation. 2008;68(SUPPL. 241):57–63. 10.1080/00365510802149887 18569966

[2] Peake M, Whiting M. Measurement of serum creatinine—current status and future goals. The Clinical biochemist Reviews / Australian Association of Clinical Biochemists. 2006;27(4):173–84. Epub 2007/06/22. ; PubMed Central PMCID: PMCPmc1784008. 17581641 PMC1784008

[3] Myers GL, Miller WG, Coresh J, Fleming J, Greenberg N, Greene T, et al Recommendations for improving serum creatinine measurement: a report from the Laboratory Working Group of the National Kidney Disease Education Program. Clin Chem. 2006;52(1):5–18. Epub 2005/12/08. 10.1373/clinchem.2005.0525144 . 16332993

[4] Killeen AA, Ashwood ER, Ventura CB, Styer P. Recent trends in performance and current state of creatinine assays. Archives of Pathology and Laboratory Medicine. 2013;137(4):496–502. 10.5858/arpa.2012-0134-CP 23544939

[5] Greenberg N, Roberts WL, Bachmann LM, Wright EC, Dalton RN, Zakowski JJ, et al Specificity characteristics of 7 commercial creatinine measurement procedures by enzymatic and jaffe method principles. Clinical Chemistry. 2012;58(2):391–401. 10.1373/clinchem.2011.172288 22166253

[6] Cobbaert CM, Baadenhuijsen H, Weykamp CW. Prime time for enzymatic creatinine methods in pediatrics. Clinical Chemistry. 2009;55(3):549–58. 10.1373/clinchem.2008.116863 19168555

[7] Drion I, Cobbaert C, Groenier KH, Weykamp C, Bilo HJ, Wetzels JF, et al Clinical evaluation of analytical variations in serum creatinine measurements: Why laboratories should abandon Jaffe techniques. BMC Nephrology. 2012;13(1). 10.1186/1471-2369-13-133 PMC3504563 23043743

[8] Panteghini M. Enzymatic assays for creatinine: time for action. Scand J Clin Lab Invest Suppl. 2008;241:84–8. Epub 2008/08/21. 10.1080/00365510802149978 . 18569972

[9] Abbott_Laboratories. Reagent Price List 2014 [5/23/2014].

[10] Horvath AR, Lord SJ, StJohn A, Sandberg S, Cobbaert CM, Lorenz S, et al From biomarkers to medical tests: The changing landscape of test evaluation. Clin Chim Acta. 2014;427(0):49–57. 10.1016/j.cca.2013.09.018. 24076255

[11] Yoe C. Principles of risk analysis: decision making under uncertainty Primer on Risk Analysis: CRC Press; 2011 p. 1–25.

[12] Qiu L, Guo X, Zhu Y, Shou W, Gong M, Zhang L, et al Effect of picric acid and enzymatic creatinine on the efficiency of the glomerular filtration rate predicator formula. Clin Lab. 2012;59(5–6):511–22. 10.7754/clin.lab.2012.120524 23865349

[13] College of American Pathologists. Chemistry/Therapeutic Monitoring, Participant Survey. 2014.

[14] Schmidt RL, Kordy MA, Genzen JR, Straseski JA, Greene DN, Lehman CM. A mathematical procedure to estimate the impact of a change in method on discordance or misclassification at a decision limit in laboratory method comparison studies. Clin Chim Acta. 2015;440:23–30. 10.1016/j.cca.2014.10.043 25444744

[15] Fraser CG. Biological Variation: From Principles to Practice: AACC Press; 2001.

[16] Selvin E, Juraschek SP, Eckfeldt J, Levey AS, Inker LA, Coresh J. Within-person variability in kidney measures. Am J Kidney Dis. 2013;61(5):716–22. 10.1053/j.ajkd.2012.11.048 23337799 PMC3628297

[17] Badrick T, Turner P. The Uncertainty of the eGFR. Indian Journal of Clinical Biochemistry. 2013;28(3):242–7. 10.1007/s12291-012-0280-1 24426218 PMC3689324

[18] Reinhard M, Erlandsen EJ, Randers E. Biological variation of cystatin C and creatinine. Scandinavian Journal of Clinical and Laboratory Investigation. 2009;69(8):831–6. 10.3109/00365510903307947 19929276

[19] Klee GG, Schryver PG, Saenger AK, Larson TS. Effects of analytic variations in creatinine measurements on the classification of renal disease using estimated glomerular filtration rate (eGFR). Clinical Chemical Laboratory Medicine. 2007;45(6):737–41. 10.1515/CCLM.2007.168 17579525

[20] Tholen DW, Kallner A, Kennedy JW, Krouwer JS, Meier K. Evaluation of Precision Performaance of Quantitative Measurement Methods; Approved Guideline—Second Edition. Evaluation. 2004;24(25).

[21] Levey AS, Stevens LA, Schmid CH, Zhang Y, Castro Iii AF, Feldman HI, et al A new equation to estimate glomerular filtration rate. Annals of Internal Medicine. 2009;150(9):604–12. 19414839 10.7326/0003-4819-150-9-200905050-00006 PMC2763564

[22] Stevens LA, Coresh J, Feldman HI, Greene T, Lash JP, Nelson RG, et al Evaluation of the modification of diet in renal disease study equation in a large diverse population. J Am Soc Nephrol. 2007;18(10):2749–57. Epub 2007/09/15. 10.1681/asn.2007020199 . 17855641

[23] Inker LA, Astor BC, Fox CH, Isakova T, Lash JP, Peralta CA, et al KDOQI US commentary on the 2012 KDIGO clinical practice guideline for the evaluation and management of CKD. Am J Kidney Dis. 2014;63(5):713–35. Epub 2014/03/22. 10.1053/j.ajkd.2014.01.416 . 24647050

[24] K/DOQI clinical practice guidelines for chronic kidney disease: evaluation, classification, and stratification. Am J Kidney Dis. 2002;39(2 suppl 1):S1–266. 11904577

[25] National_Kidney_Foundation. KDIGO Clinical Practice Guideline for the Evaluation and Management of Chronic Kidney Disease. Kidney inter, Suppl. 2013;3(1):1–150.

[26] Go AS, Chertow GM, Fan D, McCulloch CE, Hsu C-y. Chronic Kidney Disease and the Risks of Death, Cardiovascular Events, and Hospitalization. New England Journal of Medicine. 2004;351(13):1296–305. 10.1056/NEJMoa041031 . 15385656

[27] James PA, Oparil S, Carter BL, Cushman WC, Dennison-Himmelfarb C, Handler J, et al 2014 evidence-based guideline for the management of high blood pressure in adults: report from the panel members appointed to the Eighth Joint National Committee (JNC 8). JAMA. 2014;311(5):507–20. 10.1001/jama.2013.284427 24352797

[28] Sim JJ, Rutkowski MP, Selevan DC, Batech M, Timmins R, Slezak J, et al Kaiser Permanente Creatinine Safety Program: A Mechanism to assure widespread detection and care for chronic kidney disease. The American journal of medicine. 2015. 10.1016/j.amjmed.2015.05.037 26087046

[29] Schmidt RL, Kordy MA, Genzen JR, Straseski JA, Greene DN, Lehman CM. A mathematical procedure to estimate the impact of a change in method on discordance or misclassification at a decision limit in laboratory method comparison studies. Clin Chim Acta. 2015;440(0):23–30. 10.1016/j.cca.2014.10.043. 10.1016/j.cca.2014.10.043 25444744

