# Supplementary figures and images for: A Risk Assessment of the Jaffe vs Enzymatic Method for Creatinine Measurement in an Outpatient Population
Source: PLoS One. 2015 Nov 24;10(11):e0143205. doi: 10.1371/journal.pone.0143205 (PMC4657986; doi:10.1371/journal.pone.0143205)

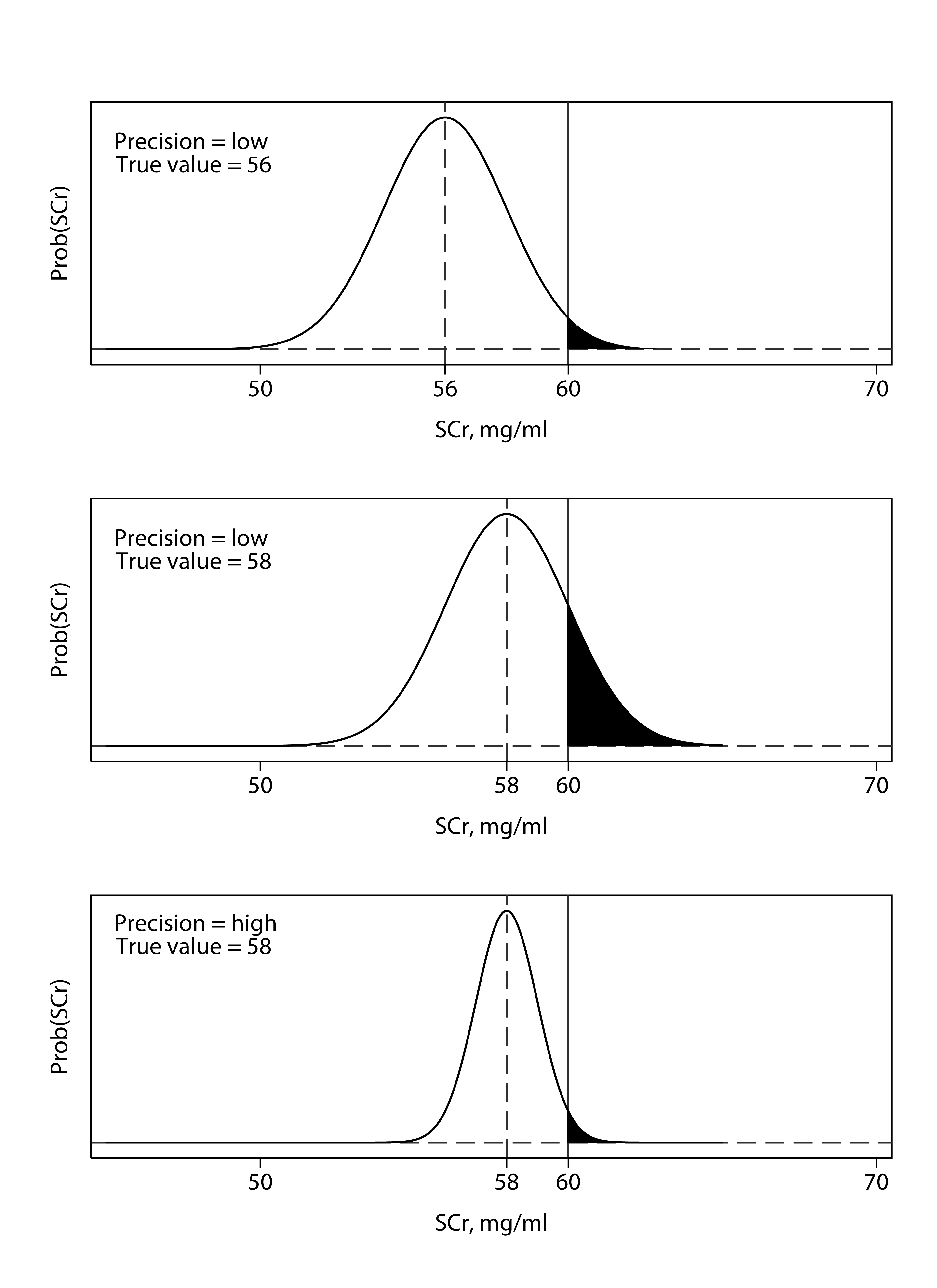

Supplement: S1 Fig — Each panel shows the distribution of measurements centered on the true value. The width of the distribution depends on the variation of the measurement. The decision limit is 60 for all three cases. The shaded area indicates probability of misclassification given the distance between the true value and decision limit and the precision. (TIF) [file pone.0143205.s001.tif]

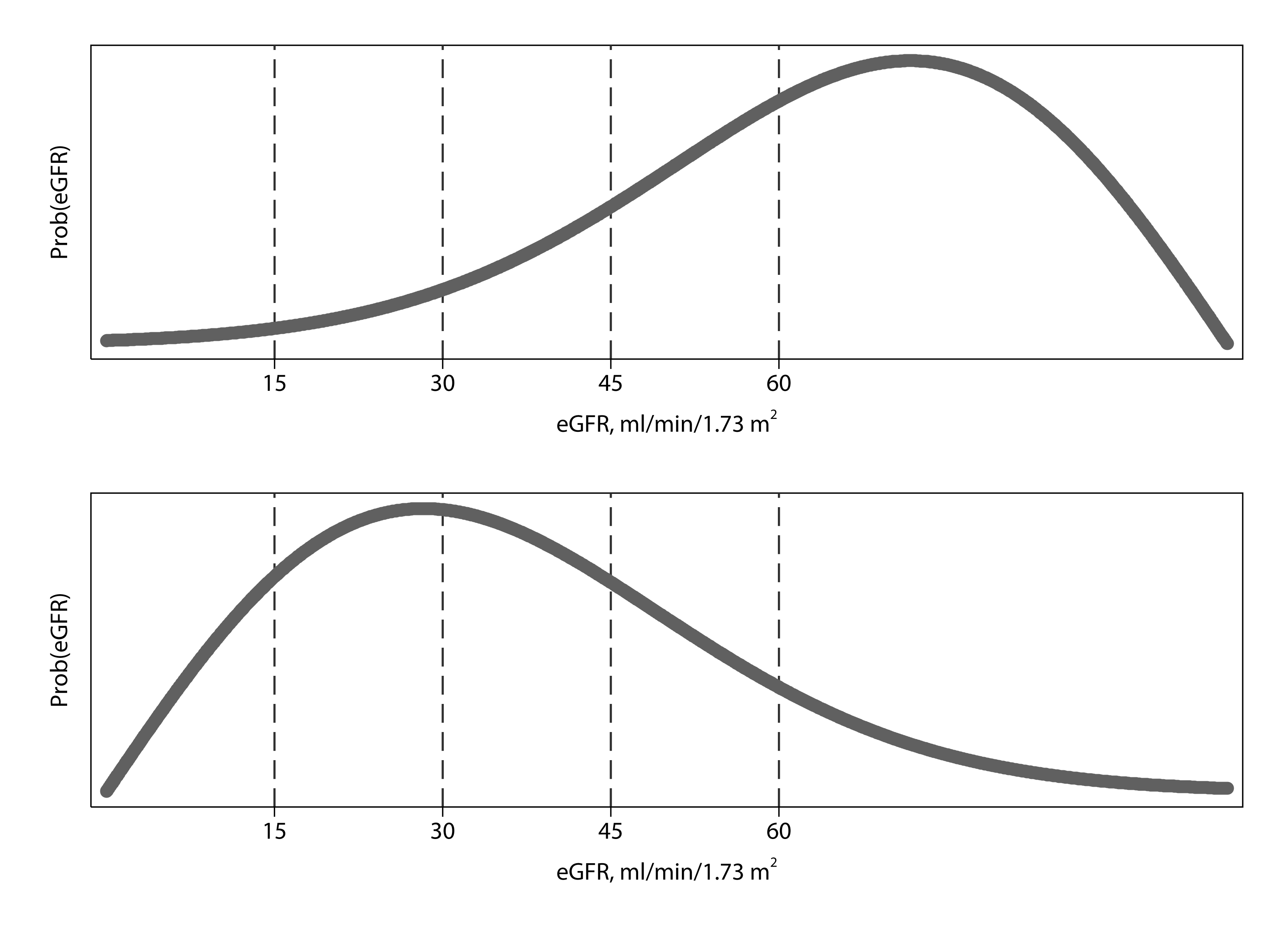

Supplement: S2 Fig — The graph shows the eGFR distribution for two different hypothetical patient populations. The upper panel represents a relatively healthy population in which the majority of eGFR values are greater than 60 ml/min/1.73 m2. In this population, the misclassification rate will be greatest at 60 ml/min/1.73 m2. The lower panel represents a specialized population with kidney disease. In this population, the misclassification rate will be greatest at 30 ml/min/1.73 m2. (TIF) [file pone.0143205.s002.tif]

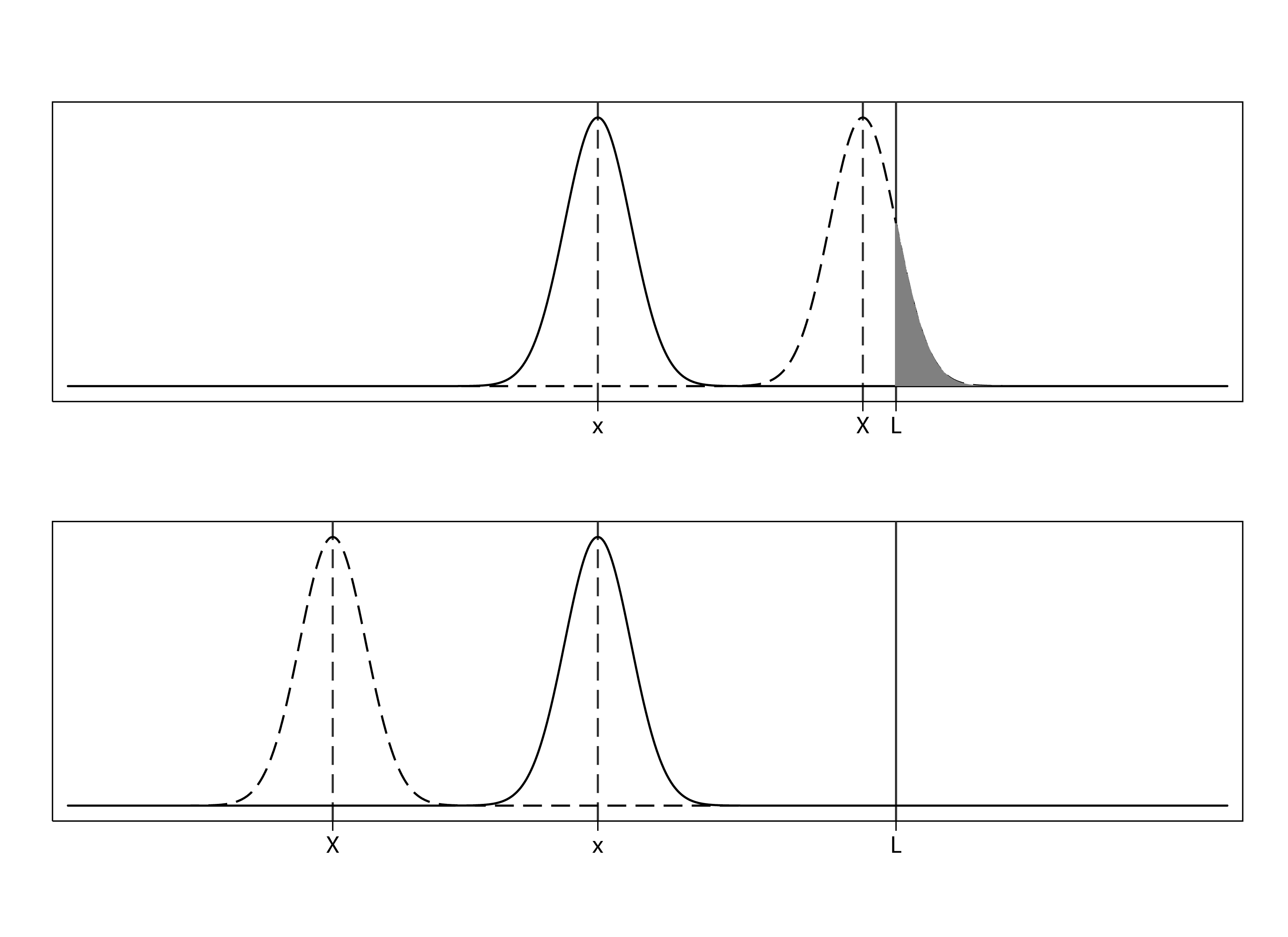

Supplement: S3 Fig — The true result is indicated by a lower case x and the biased value is indicated by an upper case X. The decision limit is indicated by L. Due to imprecision, the observed values will form a distribution around the mean value. In the upper panel, bias moves the mean toward the decision limit and increases the probability of misclassification. In the lower panel, bias moves the mean away from the decision limit and decreases the probability of misclassification. (TIF) [file pone.0143205.s003.tif]

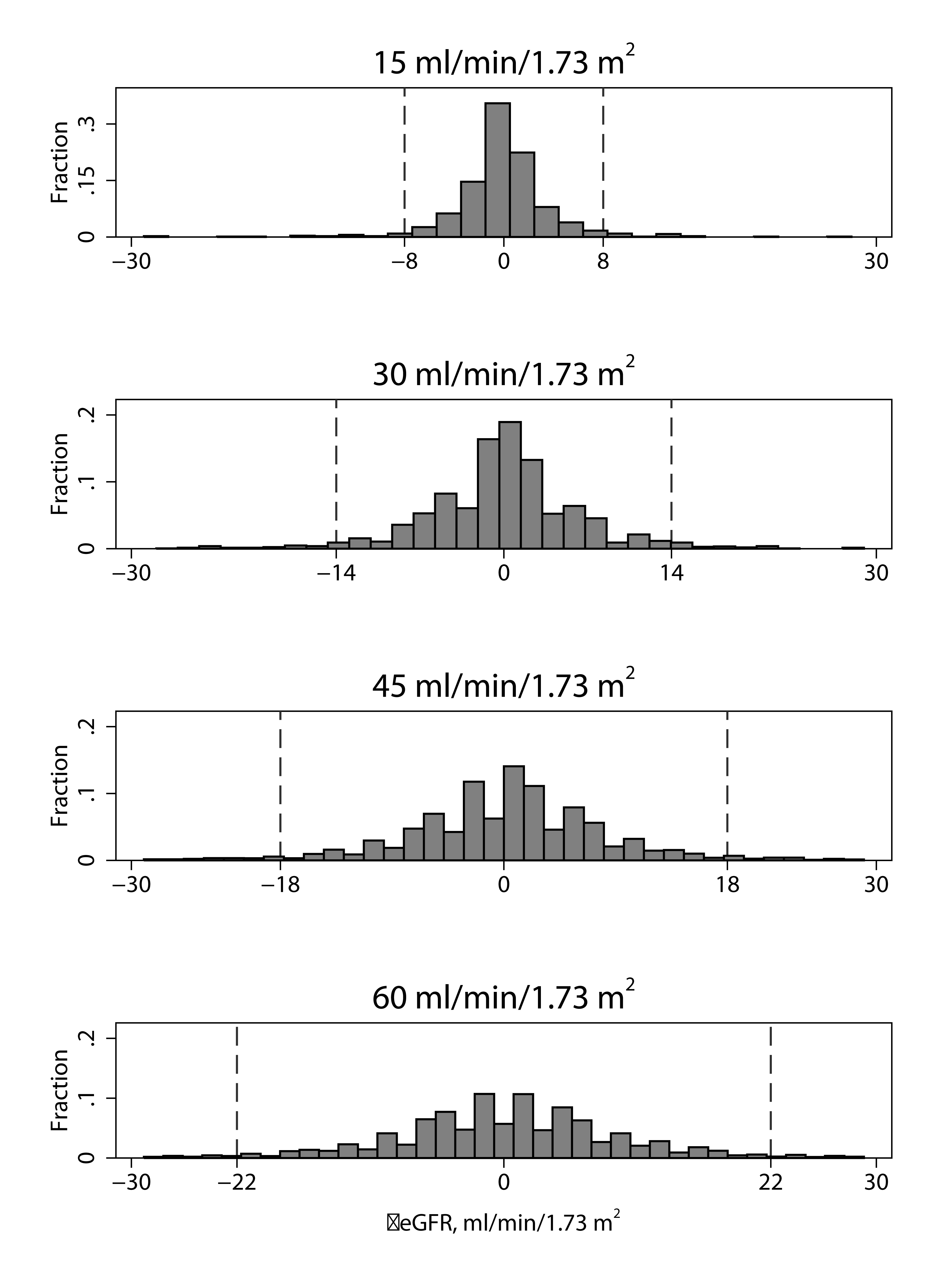

Supplement: S4 Fig — The lower limit is the 2.5th percentile and the upper limit is the 97.5th percentile of observations. (TIF) [file pone.0143205.s004.tif]

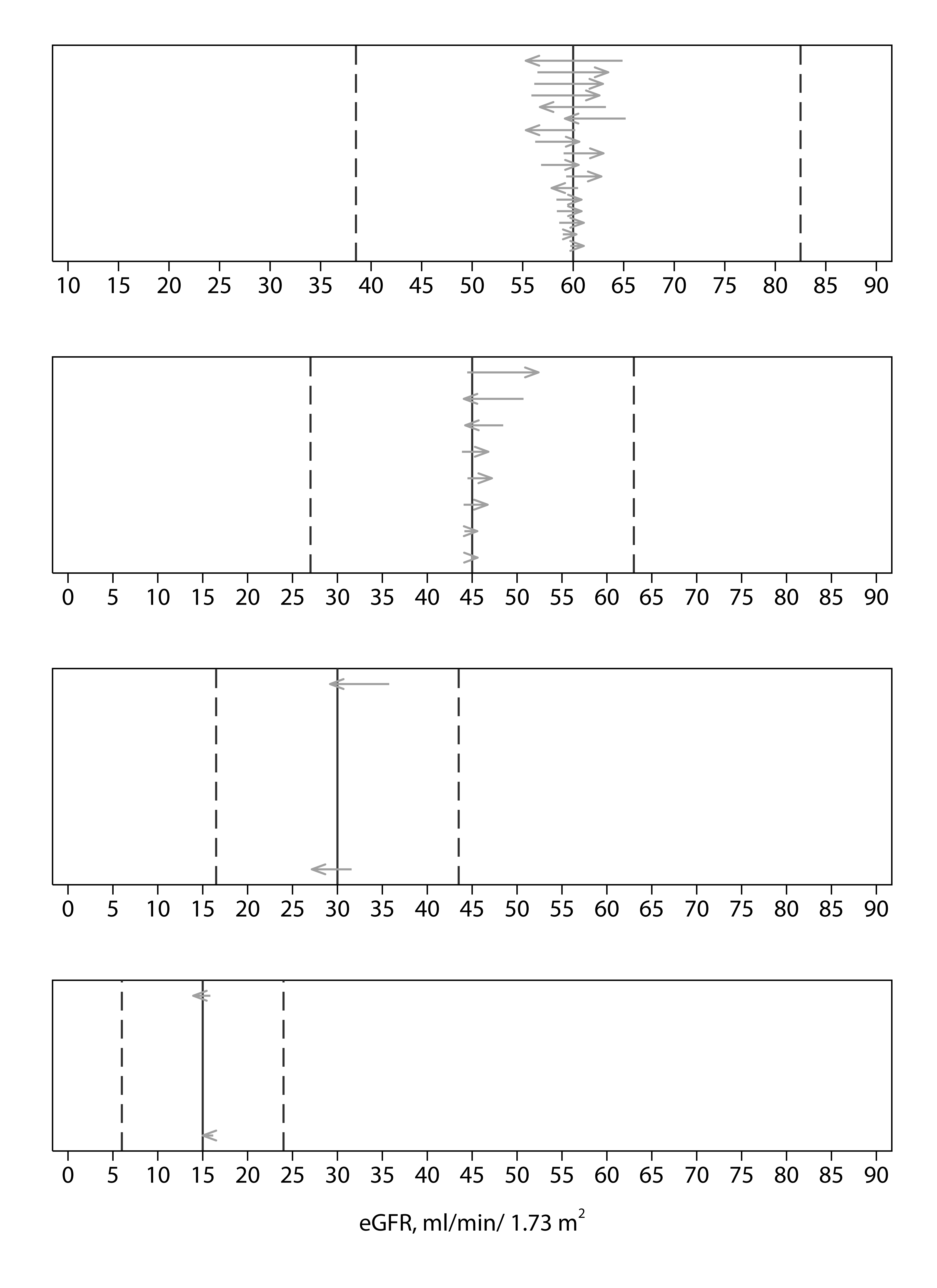

Supplement: S5 Fig — Each line represents the difference between the Jaffe result and enzymatic result. The vertical lines indicate the decision limit and two standard deviations of the difference (Jaffe-enzymatic) due to measurement imprecision at the decision limit. The arrows are directed from the enzymatic result toward the Jaffe result (the Jaffe result is greater than enzymatic when arrows point from left to right). Heavy lines indicate statistically significant discordances (i.e., greater than two standard deviations of the biologic variation) and light lines indicate nonsignificant discordances (there are no heavy lines in this graph). (TIF) [file pone.0143205.s005.tif]
